# A spatiotemporal simulation study on the transmission of harmful microorganisms through connected healthcare workers in a hospital ward setting

**DOI:** 10.1186/s12879-021-05954-7

**Published:** 2021-03-12

**Authors:** J. M. van Niekerk, A. Stein, M. H. E. Doting, M. Lokate, L. M. A. Braakman-Jansen, J. E. W. C. van Gemert-Pijnen

**Affiliations:** 1 Department of Psychology, Health and Technology/Center for eHealth Research and Disease Management, Faculty of Behavioural Sciences, University of Twente, Enschede, The Netherlands; 2 Department of Earth Observation Sciences, Faculty of Geo-Information Science and Earth Observation (ITC), University of Twente, Enschede, The Netherlands; 3 Department of Medical Microbiology, University of Groningen, University Medical Center Groningen, Groningen, The Netherlands

**Keywords:** Spatiotemporal risk, RFID, Wearable proximity sensors, Spatiotemporal simulation, Healthcare-associated infections, Transmission, Hand hygiene compliance

## Abstract

**Background:**

Hand transmission of harmful microorganisms may lead to infections and poses a major threat to patients and healthcare workers in healthcare settings. The most effective countermeasure against these transmissions is the adherence to spatiotemporal hand hygiene policies, but adherence rates are relatively low and vary over space and time. The spatiotemporal effects on hand transmission and spread of these microorganisms for varying hand hygiene compliance levels are unknown. This study aims to (1) identify a healthcare worker occupancy group of potential super-spreaders and (2) quantify spatiotemporal effects on the hand transmission and spread of harmful microorganisms for varying levels of hand hygiene compliance caused by this group.

**Methods:**

Spatiotemporal data were collected in a hospital ward of an academic hospital using radio frequency identification technology for 7 days. A potential super-spreader healthcare worker occupation group was identified using the frequency identification sensors’ contact data. The effects of five probability distributions of hand hygiene compliance and three harmful microorganism transmission rates were simulated using a dynamic agent-based simulation model. The effects of initial simulation assumptions on the simulation results were quantified using five risk outcomes.

**Results:**

Nurses, doctors and patients are together responsible for 81.13% of all contacts. Nurses made up 70.68% of all contacts, which is more than five times that of doctors (10.44%). This identifies nurses as the potential super-spreader healthcare worker occupation group. For initial simulation conditions of extreme lack of hand hygiene compliance (5%) and high transmission rates (5% per contact moment), a colonised nurse can transfer microbes to three of the 17 healthcare worker or patients encountered during the 98.4 min of visiting 23 rooms while colonised. The harmful microorganism transmission potential for nurses is higher during weeknights (5 pm – 7 am) and weekends as compared to weekdays (7 am – 5 pm).

**Conclusion:**

Spatiotemporal behaviour and social mixing patterns of healthcare can change the expected number of hand transmissions and spread of harmful microorganisms by super-spreaders in a closed healthcare setting. These insights can be used to evaluate spatiotemporal safety behaviours and develop infection prevention and control strategies.

**Supplementary Information:**

The online version contains supplementary material available at 10.1186/s12879-021-05954-7.

## Background

The majority of healthcare-associated infections are caused by direct or indirect transmission of *Enterococcus faecium, Staphylococcus aureus, Klebsiella pneumoniae, Acinetobacter baumannii, Pseudomonas aeruginosa* or *Enterobacter spp.* (ESKAPE) [1]. These harmful microorganisms (HMO) can survive on human skin and hospital surfaces for extended periods and lead to high cross-transmission rates between healthcare workers (HCW) and patients [2–4]. The ease of transmission of HMO depends upon the features of the microorganism, patient characteristics and the behaviour of healthcare workers (HCW), whereas the damage caused by the infection that follows ranges from none to potentially fatal [5].

The most effective precautionary measure to combat hand transmission and spread of harmful microorganisms in closed healthcare settings is the adherence to well established and effective hand hygiene policies also known as hand hygiene compliance (HHC) [6]. Unfortunately, HHC is often unsatisfactory with highly variable levels within and between hospitals. Rates of hand hygiene compliance range from 5 to 81%, with average compliance of approximately 40% [7]. With a level of 80% adherence seen as high levels of HHC and 95% as very high, it is not surprising that the spread of HMO in closed healthcare settings remains a major dilemma [7]. Reasons for hand hygiene non-compliance include increased work intensity, lack of education and ineffective placement or defective alcohol dispensers. For instance, 1 h of overtime worked by an HCW can lead to a 3% decrease in the level of HHC [8, 9]. The result is a highly variable level of HHC within closed healthcare settings. Compounding the non-adherence to hand hygiene policies is that the medium and method used for hand hygiene are not 100% efficient. The efficacy of hand rubbing using alcoholic rub was compared with handwashing using antibacterial soap during routine patient care [10]. Some of the patients had methicillin-resistant *Staphylococcus aureus* (MRSA). The study estimated an efficacy rate of 83% (interquartile range 78–92%) for alcoholic rub compared to 58% (interquartile range − 58 - 74%) for antibacterial soap. Even though alcoholic rub significantly outperforms antibacterial soap, some HMO may remain on the hands of the HCWs and lead to further transmissions.

The combination of colonised and uncolonized HCWs or patients, who are potentially immunocompromised and in a confined space, makes healthcare facilities a high-risk environment for the spread of HMO. The term super-spreader is used to categorise an individual with a disproportionately high potential to spread HMO. Super-spreaders were the cause of several super-spreading events (SSE) in the past with devastating consequences [11]. Highly connected HCWs can increase the risk of SSE in closed healthcare environments. The amount of contact between HCWs and patients and HHC while performing regular duties are critical factors that contribute to the extent and severity of an SSE [12].

For these reasons, the SSE is affected by the joint spatiotemporal behaviour, i.e. the where and when, by the social mixing patterns, i.e. with whom of the HCW or patients inside a hospital ward, and by the level of HHC, including its variability. Therefore, it is necessary to understand the spatiotemporal effect on the hand transmission and spread of HMO for varying levels of HHC for potential super-spreaders in a closed healthcare setting.

Healthcare institutes are now adopting automatic contact tracking methods like Radio Frequency Identification (RFID) technology by tagging healthcare equipment, HCWs and patients to improve logistics and patient safety. There is still a reluctance to fully adopt this technology, mainly driven by security and privacy concerns [13]. Real contact data between patient and HCWs became more prevalent since 2002 when data were collected using shadowing. Medical records, surveys and sensors became more important for data and contact detection. Assab et al. (2017) showed that studies using empirical contact data within closed healthcare settings lead to a better understanding of the transmission and spread of HMO. Such data can result in the development of improved control interventions. Using real-time RFID tracking data, it is possible to model the spread of HMO at an individual level rather than using a compartmental-based model [5, 14]. RFID data have been used to model the spread of HMO in different closed healthcare settings and at different proximities using a temporal proximity network at schools [15, 16], conferences [17], households [18], hospitals [19–25] and other healthcare facilities [26]. In addition to recent research, data collection and modelling innovations are needed to implement better control strategies [27]. Studies based upon contact data only are unable to determine the effect of spatiotemporal healthcare policies like HHC. A few hours of RFID tracking can be sufficient to develop and calibrate a statistical model that shows the heterogeneity of spatiotemporal social contact patterns, representing how people socially interact in space and time [22].

The spatiotemporal effects of varying levels HHC on the transmission and spread of hand-transmitted HMO in a closed healthcare setting must still be quantified, based upon empirical spatiotemporal tracking data. HCWs and policymakers may benefit from understanding the impact of spatiotemporal infection control interventions and healthcare policies on the transmission and spread of HMO.

This study’s objectives were to (1) identify an HCW occupancy group of potential super-spreaders and (2) quantify the spatiotemporal effects on the transmission and spread of HMO for varying levels of hand hygiene compliance caused by this group.

## Methods

**Fig. 1:**
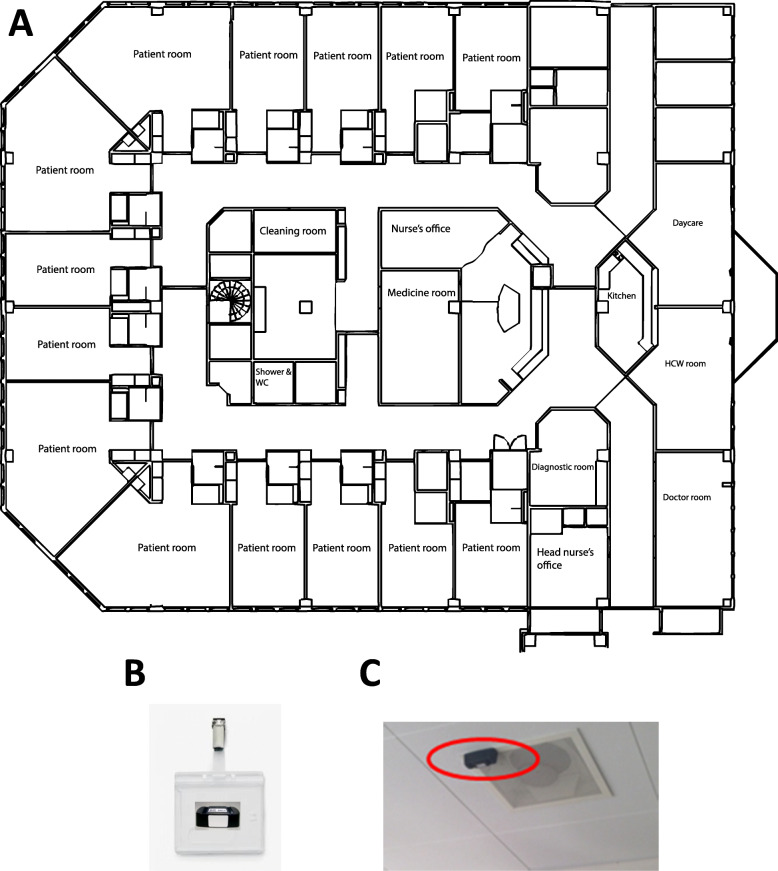
Floorplan of the 32-bed general hospital ward, for stomach, gut and liver patients. **a** = Floorplan of the 32-bed general hospital ward, for stomach, gut and liver patients where sample data were collected using **b** = RFID tags worn by HCWs during data collection using **c** = RFID readers placed on the ceiling of the rooms inside the ward

We used spatiotemporal data from the University Medical Center Groningen (UMCG), one of the largest hospitals in the Netherlands with more than 10,000 employees and almost 1400 beds. Between 2 April 2018 and 8 April 2018, data were collected in a 32-bed general hospital ward, for stomach, gut and liver patients (Fig. 1: a). The dates were chosen such that they cover a full calendar week from Monday to Sunday and all shifts to increase the representativeness of the parameter estimates. The ward’s floor plan was divided into 33 rooms of which 14 were patient rooms, with between one and four beds, eight storage areas and ten other rooms, including a doctor’s office and a medicine room. All facilities are located in the centre of the ward to minimise distances to crucial parts of the ward, including the rinsing kitchen and medication rooms. Single patient rooms are near the entrance of the ward to enable easy isolation of potentially infectious patients.

Data were collected using RFID sensors worn by the HCWs working in the ward during the study period. Seven HCW occupation groups were identified, namely *doctor*, *nurse*, *cleaner*, *department assistant*, *department co-assistant*, *consultant* and *feeding assistant*. The RFID tags were assigned to specific occupation groups. HCWs randomly selected an RFID tag at the start of their shift according to their occupation. The RFID tags (Fig. 1: b) emit radio signals with unique identifying information and RFID readers (Fig. 1: c), on the ceiling of the rooms, register those signals. The RFID reader’s range was set to the size of the rooms and they continuously monitored the uniquely identifiable RFID tags in their range. HCWs moving in and out of the rooms were registered and the data were generated and stored. The data consist of a room ID, an RFID sensor ID and a DateTime stamp corresponding to the RFID tag movement into and out of a room (Table 1). The spatial resolution is at the room level and is defined by the set of rooms inside the ward. The temporal resolution equals the second at which the observation signal was received.

**Table 1:** Example of data collected using the RFID sensors and readers

Room	From	To	Sensor
54.3.35A	03/04/2018 16:49	03/04/2018 16:50	58,007
54.3.45	03/04/2018 16:51	03/04/2018 17:00	58,007
54.3.14	03/04/2018 17:00	03/04/2018 17:37	58,007
54.3.17	03/04/2018 17:37	03/04/2018 17:41	58,007

The sampled data were divided into two subsets. Data in subset 1 contained the sampled data collected during weekdays (7 am – 5 pm) and data in subset 2 those collected during the evenings (5 pm – 7 am) and over the weekend.

Contact data were extracted from the empirical spatiotemporal data. They are generated by the underlying contact network between HCWs and determines the possible pathways over which the spread of HMO occurs over space and time [28, 29]. Since the spatial resolution of the collected data was at the room level and not at the face-to-face level, an assumption was needed for the contact definition. Depending upon the data collection context, co-occurrence data can serve as a proxy for face-to-face contact data [30]. In this study, co-occurrence can occur inside the limited space defined by the ward rooms, increasing the probability of HCWs to enter the face-to-face close range proximity (1.5 m) of other HCW or patients. For this reason, we define a contact as the physical co-occurrence of two HCWs or a HCW and a patient in a ward room. For example, if an HCW enters a patient room, then the HCW and the patient are assumed to be in contact with each other for the time over which they co-occur in that room.

A guideline to identify super-spreaders is to identify the 20% of the people contributing to at least 80% of the transmission potential [31]. We define the transmission potential as the number of 30-s intervals (*contact moment*) of contact with other HCWs or patients. The transmission potential is estimated for all HCW occupation groups and compared to identify disproportionality and thus potential super-spreaders [Additional file 1].

We estimated the effect of the transmission and spread of an HMO by a colonised HCW from the potential super-spreading occupation group for varying levels of HHC defining five risk outcomes. The risk outcomes are defined in five variables: the amount of time (minutes) spent colonised (RO1), the amount of time (minutes) spent with HCWs or patients (RO2), the number of HCWs or patients encountered (RO3), the number of transitions made from one room to another (RO4) and the expected number of HMO transmissions to other HCWs or patients before successfully performing hand hygiene (RO5).

**Fig. 2:**
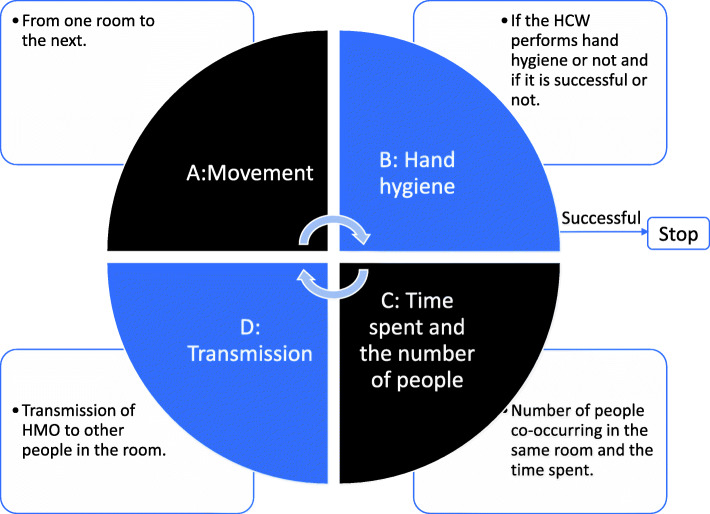
The four-part of the simulation workflow. **a** and **c** depend upon the sampled data and (**b)** and (**d)** on initial assumptions from literature. The simulation ends when the HCW successfully performs hand hygiene in part B

To estimate RO1 – RO5, we constructed a dynamic agent-based transition simulation model [32]. To simulate the underlying distribution from the sampled data, we first estimated this distribution, followed by resampling to generate more samples. This simulation process aids us to explore the consequences of initial simulation assumptions. The simulation follows a four-part (A-D) workflow (Fig. 2). We assumed that RO1 – RO5 depend upon the order in which an HCW moves between the different rooms in the hospital ward (A), the likelihood of the HCW performing hand hygiene and the efficacy of doing so (B), the amount of time an HCW spends in each room with other HCWs or patients (C) and the transmission dynamics of the HMO (D). Parts A and C are based on statistics from the sampled data, while parts B and D are based on assumptions from the literature.

For part A in the simulation workflow, we used continuous Markov chains. They allowed us to model the movement of HCWs from one of the *n* rooms to the next [33]. If *R* is the set of *n* rooms, i.e. *R* = {*R*_1_, *R*_2_, …, *R*_*n*_}, then the transition probability *p*_*ij*_ (Formula 1) in row *i* and column *j* of the *n* × *n* transition probability matrix ***P*** (Formula 2) is the probability that an HCW will transit from room *R*_*i*_ to room *R*_*j*_ during the next transition. Since an HCW will either stay in the same room or move to another room after the next transition, the rows of the matrix ***P*** add up to 1 i.e. $\sum \limits_{j=1}^n{p}_{ij}=1$ for *i* = 1, …, *n*. Each element *p*_*ij*_ is between 0 and 1 inclusively, i.e. 0 ≤ *p*_*ij*_ ≤ 1 for *i*, *j* ∈ (1, …, *n*). An estimate for *p*_*ij*_ is obtained by dividing the number of transitions from *R*_*i*_ to *R*_*j*_ by the total number of transitions from *R*_*i*_. Using only the transition data of the potential super-spreading occupation group, we obtain a transition probability matrix ***P***.

$$ {p}_{ij}=P\left(\mathrm{Next}\ \mathrm{room}={R}_j|\mathrm{Current}\ \mathrm{room}={R}_i\right)\ \mathrm{for}\ \mathrm{i},\mathrm{j}\in \left(1,\dots, \mathrm{n}\right)\ \mathrm{Formula}\ 1 $$

$$ \boldsymbol{P}={\displaystyle \begin{array}{c}\ {R}_1\kern0.5em \dots \kern0.75em {R}_n\\ {}\begin{array}{c}{R}_1\\ {}\vdots \\ {}{R}_n\end{array}\left[\begin{array}{ccc}{p}_{11}& \cdots & {p}_{1n}\\ {}\vdots & \ddots & \vdots \\ {}{p}_{n1}& \cdots & {p}_{nn}\end{array}\right]\end{array}}\ \mathrm{Formula}\ 2 $$

We assume that the length of time spent in each room (${\psi}_{R_i}$) is exponentially distributed with parameter *η* with mean 1/*η* and variance 1/*η*^2^ [29]. The estimated values of *η* and the average number of HCWs or patients co-occurring inside each room, together with the corresponding estimated variance, are obtained at room level from the sampled data. We assumed that the number of HCWs or patients co-occurring within each room follows either a Gaussian distribution or a Poisson distribution with mean and standard deviation equal to the estimates obtained from the sampled data. Since no patient location data were available, we assumed that all patient rooms are occupied.

The performance and efficacy of hand hygiene (B) compliance and the transmission of an HMO (C) are simulated using agent-based modelling and the corresponding model assumptions in Table 2, based upon a study by Thomas Hornbeck et al. [11].

**Table 2:** Agent-based model parameters (Thomas Hornbeck et al.)

Symbol	Definition	Range
*P*	Probability of transmission per 30 s of contact	0.0005, 0.005 and 0.05
*λ*	Hand hygiene efficacy using alcohol rub	0.83
*γ*	Hand hygiene compliance level	*μ* = 0.05,0.25,0.5,0.75,0.95
		and *σ* = 0.1

The simulation starts with one colonised HCW from the potential super-spreading occupation group in a random room inside the hospital ward. It ends when the HCW successfully performed hand hygiene. [Additional file 1] One thousand simulations were performed for the three different rates of transmission (*P*) for each of the five HHC distributions. The result of the simulation consists of 15 (3 × 5) scenarios with outputs RO1 – RO5. The simulations were repeated for the subsets 1 and 2, both separately and combined.

We summarise the simulation assumptions made by the workflow section as follows:

Workflow A: Movement

1. Contact definition is based upon HCWs or patients co-occurring in the same room.
2. Patient rooms are always occupied by at least 1 patient, being the reason for the HCW to visit the room.

Workflow B: Hand hygiene

1. A colonised HCW can perform hand hygiene once during every transition between rooms.
2. For a colonised HCW to be decolonized, hand hygiene needs to be performed and it needs to be successful. The former depends upon the action of the HCW with probability *γ* and the latter on the efficacy of the solution used to perform hand hygiene with probability *λ*.

Workflow C: Time spent and Number of people

1. The number of minutes an infected HCW spends in a room *R*_*i*_ is given by ${\psi}_{R_i}\sim Exp\left(\frac{1}{\eta}\right)$, where η is the sample average of ${\psi}_{R_i}$.
2. The number of HCWs or patients co-occurring in room *R*_*i*_ with the infected HCW is given by ${\omega}_{R_i}\sim N\left(\nu, {\varphi}^2\right)$ or ${\omega}_{R_i}\sim Poisson\left(\nu \right)$, where *ν* and *φ*^2^ are estimated using the average and variance of ${\omega}_{R_i}$ from the sampled data respectively.

Workflow D: Transmission

1. Only colonised nurses can transmit an HMO.
2. HCW and patients only have two states: susceptible and colonised.
3. Number of colonised HCW or patients after co-occurring with a colonised HCW for *m* contact moments with a probability of transmission *P* for each 30 s of co-occurrence is distributed as *I*~*Bin*(*m*, *P*).

There are two key moments in the model. The first key moment is when the colonised HCW enters a room – that is when an opportunity is given to perform hand hygiene with probability *γ*, corresponding to part B of the simulation workflow. Five probability distributions are used to simulate HHC for simulation. A Gaussian distribution with mean 0.05 represents very low HHC, while means equal to 0.25 and 0.5 show the effect of low to average HHC and 0.75 to 0.95 for high to near-perfect HHC levels, respectively. Should colonised HCW perform hand hygiene, then the probability that the hand hygiene was successfully performed, meaning that all traces of the HMO were eradicated, equals *λ*. The probability of successful use of hand hygiene is based upon Girou et al. [10] and the different compliance levels (low, medium and high) are based upon Temime et al. [12].

The second key moment is when more than one HCW or patient co-occurs with *g* in the same room. The colonised HCW has a probability of transferring microbes to all HCWs or patients in the room every 30 s with probability *P*. The probability of transmitting an HMO from one person to another, results in a probability equal to 1.5–13.5% of transmission for every 15 min spent together [12]. We assume that transmissions between all HCWs and patients are equally likely for each contact moment in the same room. For example, HCWs or patients in contact with a potential super-spreader will be subject to the probability stated in Formula 3 during the first contact moment. The last two terms in Formula 1 decrease the probability of transmission because of the chance that the potential super-spreader will effectively perform hand hygiene and not carry the HMO anymore. Only the parameter *P* remains for subsequent contact moment because the potential super-spreader only performs hand hygiene when entering the room.

$$ P\left[ Susceptible\to Infected|n=1\right]=P\times \gamma \times \left(1-\lambda \right)\ \mathrm{Formula}\ 3 $$

For successive contact moments, we assume that the probability that a colonised HCW transfers microbes to an uncolonized HCW or patient follows a binomial distribution with parameter *m* indicating the number of contact moments and parameter *P* indicating the probability of transmission. To model this as a binomial distribution, we assume that there are only two outcomes, i.e. colonised and uncolonized, that each contact moment is independent of the other and that the probability of transmission stays constant.

The effect of *P* is positively correlated with the number of transmissions, meaning that more transmissions should take place if the rate of infection increases. However, model parameters *λ* and *γ* have an inverse relationship with the expected number of transmissions. Some simulation parameters are positively correlated, and some are negatively correlated with the expected number of transmissions. Opposite parameter correlations make it possible to create scenarios where the expected number of transmissions is mitigated and even entirely off-set. These scenarios provide further insight into the effects of the initial simulation assumptions propagated through the sampled spatiotemporal data.

## Results

During the 7 days of data collection, a total of 2631 observations were recorded of which 58 had to be removed because of spurious measurements detected using outlier detection and identifying aberrant movement patterns in the collected data. During the 7 days, 2432 co-occurrences were derived from the 2573 sampled observations which equate to 504 h (30 272 min) of contact data. Nurses and doctors were together responsible for 81.13 and 80.19% of all contacts and time spent in contact, respectively (Table 3). Nurses made up 70.68 and 68.06% of these percentages, five times more than the second higher HCW occupation group, *doctor* (10.44 and 12.13%). Therefore, a colonised nurse has a disproportionately high potential of transmitting and HMO based on the amount of contact and time spent with HCWs or patients. For these reasons, we investigate the *nurse* HCW occupation group as potential super-spreaders in this study.

**Table 3:** The number of contacts and duration of those contacts by occupation group

(Occupation) Group	Number of Contacts (% of total)	Number of Contact Minutes (% of total)	Average of Contact Minutes (SD)
Cleaner	71 (2.9%)	443 (1.5%)	6.24 (10.27)
Co-assistant	29 (1.2%)	290 (1.0%)	10.00 (12.81)
Consultant	144 (5.9%)	2230 (7.4%)	15.49 (23.27)
Department assistant	200 (8.2%)	2953 (9.8%)	14.77 (16.17)
Doctor	254 (10.4%)	3671 (12.1%)	14.45 (20.59)
Feeding assistant	15 (0.6%)	82 (0.3%)	5.47 (8.26)
Nurse	1719 (70.7%)	20,603 (68.1%)	11.99 (20.13)
Total	2432 (100.0%)	30,272 (100.0%)	12.45 (19.80)

Individual percentages may not add up to 100% because they are rounded to the first decimal place

**Fig. 3:**
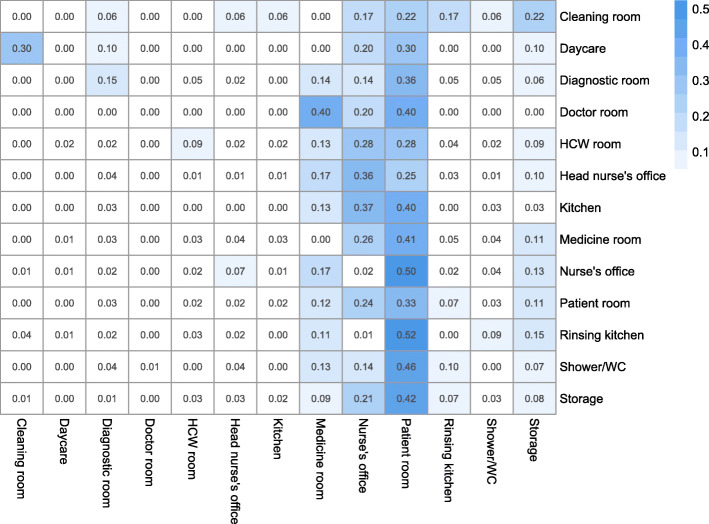
Transition probability matrix ***P*** for movement of *nurse* between ward rooms*.* The transmission probabilities are given as *p*_*ij*_ in the *i*^*th*^ row and *j*^*th*^ column for the movement of *nurse* between rooms. Each element is the estimated probability that a nurse will transition from the room *i* to room *j* after the next transition

The estimated transition probability matrix (***P***) for *nurse* summarises the transitions of *nurse* between rooms observed in the sampled data (Fig. 3). According to ***P***, *nurse* is most likely to transit to either a patient room, the medicine room or the nurse’s office.

Nurses spend the most time (19.39 m) and co-occur with the most HCWs (2.16) per visit in the nurse’s office (Table 4). For this reason, the relatively high estimated probability that a *nurse* will transit to the nurse’s office implies that an HCW of the occupation group *nurse* spends a large portion of their time here while co-occurring with a relatively large number of people. Nurses spend less time in patient rooms than the nurse’s office (11.5 m vs. 19.39 m), but the average number of people co-occurring is almost the same as in nurse’s office (2.09 vs. 2.16). Since we assumed that there is at least one patient in the patient room, the expected number of HCW and patients in contact in patient rooms is more than two by definition.

**Table 4:** Number of HCWs or patients and the time they co-occurred in each room

Room	Average number of co-occurrences (SD)	Average minutes spent co-occurring (SD)
Cleaning room	1.45 (0.4)	8.05 (12.5)
Daycare	1.10 (0.3)	8.55 (11.1)
Diagnostic room	1.11 (0.6)	27.57 (45.0)
Doctor room	1.00 (0.0)	4.82 (5.2)
HCW room	1.04 (0.3)	18.17 (22.7)
Head nurse’s office	1.82 (1.6)	18.64 (33.1)
Kitchen	1.15 (0.5)	9.51 (10.0)
Medicine room	1.16 (0.5)	10.02 (15.6)
Nurse’s office	2.16 (1.6)	19.39 (26.7)
Patient room	2.09 (0.4)	11.55 (19.0)
Rinsing kitchen	1.00 (0.1)	0.08 (0.2)
Shower/WC	1.10 (0.4)	10.74 (20.9)
Storage	1.08 (0.4)	7.73 (13.6)

Average number of co-occurrences = time weighted average number of people co-occurring in the room, Average minutes spent co-occurring = average number of minutes spent co-occurring in the room

### Simulation results

The (*P* = 0.05; *λ* = 0.05) scenario in Table 5 corresponds to the highest probability of transmission (*P*) and the lowest HHC level (*λ*). For this scenario, a colonised nurse can transit through 23 wardrooms (*RO*4 = 22.03) for more than one and a half hours (*RO*1 = 98.40 ) while making contact with 17.41 HCWs or patients (*RO*3 = 17.41), resulting in 83 contacts opportunities to transmit HMO (*RO*2 = 83.13). This scenario also resulted in the highest amount of expected transmissions (*RO*5 = 3.36). Reducing the transmission rate results in an exponential decrease in the number of expected transmissions as expected.

**Table 5:** Simulated HMO transmissions potential of a colonised nurse in a hospital ward under various assumed transmission rates and hand hygiene compliance levels

Transmission probability (*P*)	HHC (λ)	RO1: Minutes spent colonised (SD)	RO2: Contacts (SD)	RO3: People contacted (SD)	RO4: Room transitions (SD)	RO5: Expected transmissions (SD)
0.05	0.05	98.40 (73.43)	83.13 (70.69)	17.41 (13.65)	23.09 (22.03)	3.36 (2.79)
0.005	0.05	101.60 (76.20)	83.46 (69.22)	17.76 (13.50)	24.03 (22.84)	0.41 (0.34)
0.0005	0.05	98.27 (75.87)	80.56 (69.54)	17.01 (13.59)	22.66 (21.80)	0.04 (0.03)
0.05	0.25	25.24 (26.29)	21.51 (26.53)	4.21 (4.30)	4.66 (4.11)	0.86 (1.01)
0.005	0.25	25.91 (25.68)	22.11 (26.83)	4.36 (4.27)	4.73 (4.08)	0.11 (0.13)
0.0005	0.25	25.87 (26.09)	22.76 (28.74)	4.53 (4.97)	4.78 (4.40)	0.01 (0.01)
0.05	0.5	13.68 (13.29)	12.85 (17.51)	2.42 (2.67)	2.50 (2.00)	0.51 (0.65)
0.005	0.5	13.53 (12.79)	12.68 (17.13)	2.37 (2.38)	2.41 (1.76)	0.06 (0.08)
0.0005	0.5	13.01 (13.73)	13.08 (20.20)	2.37 (2.43)	2.36 (1.81)	0.01 (0.01)
0.05	0.75	9.16 (9.06)	9.43 (14.00)	1.75 (1.79)	1.66 (1.04)	0.36 (0.49)
0.005	0.75	9.12 (8.97)	9.38 (14.08)	1.68 (1.73)	1.64 (1.04)	0.04 (0.07)
0.0005	0.75	9.12 (8.88)	9.39 (14.54)	1.71 (1.73)	1.66 (1.02)	0.00 (0.01)
0.05	0.95	7.34 (6.86)	8.23 (12.66)	1.43 (1.47)	1.32 (0.65)	0.31 (0.46)
0.005	0.95	7.12 (6.71)	8.21 (12.64)	1.47 (1.50)	1.27 (0.62)	0.04 (0.06)
0.0005	0.95	7.26 (6.79)	8.57 (13.51)	1.50 (1.50)	1.29 (0.64)	0.00 (0.01)

Sampled data for three different transmission assumptions and five levels of HHC for one colonised nurse starting in a random room in the hospital ward, a hand hygiene efficacy (*γ*) of 0.83. *P*= probability of transmission, *λ*= HHC level, *RO*1= amount of time spent colonised, *RO*2= number of contact moments, *RO*3= number of HCWs or patients made contact with, *RO*4= number of transitions between hospital ward rooms, *RO*5= expected number of HMO transmissions

In the (*P* = 0.005; *λ* = 0.75) scenario, where the level of HHC is highest and the transmission probability is lowest, the expected time that a colonised NUR would spend carrying an HMO is just more than 9 min even though the alcohol rub’s effectiveness is 83%. Note that the (*P =* 0.005; *λ* = 0.05) scenario results in a similar amount of expected number of infections as the scenario where *P* = 0.05 and *λ* = 0.5 (0.41 vs. 0.51) even though he transmission probability differs by a factor of ten.

The simulation results based upon subset 1 (Table 6) show that, for the (*P* = 0.05; *λ* = 0.05) scenario, a colonised nurse is expected to spend less time colonised while transiting through the wardrooms during weekdays than during weeknights or weekends (81.46 vs. 114.30 min) even though more HCWs or patients are expected to be encountered (19.87 vs. 16.39) by the colonised nurse. Tables 6 and 7 show that the difference between the expected number of transitions by a colonised nurse for subset 1 and 2 is less than 10% for all scenarios. The difference in the expected number of transmissions between subset 1 and 2 equals 22.7% for the (*P* = 0.05, *λ* = 0.05 ) scenario and equals 66.7% for the (*P* = 0.005, *λ* = 0.95 ) scenario. These differences result from the change of spatiotemporal and social mixing patterns of the HCWs observed during the weekdays and weeknights or weekends.

**Table 6:** Simulated HMO transmissions potential of a colonised nurse in a hospital ward under various assumed transmission rates and hand hygiene compliance levels (between 7 am-5 pm on weekdays)

Transmission probability	HHC	RO1: Minutes spent colonised (SD)	RO2: Contacts (SD)	RO3: People contacted (SD)	RO4: Room transitions (SD)	RO5: Expected infections (SD)
0.05	0.05	81.46 (58.71)	73.86 (60.06)	19.87 (15.11)	23.18 (21.34)	3.16 (2.52)
0.005	0.05	79.51 (60.02)	72.85 (62.42)	19.41 (15.64)	22.82 (21.77)	0.36 (0.31)
0.0005	0.05	81.64 (59.81)	75.58 (61.32)	20.00 (15.14)	23.34 (21.97)	0.04 (0.03)
0.05	0.25	21.47 (20.41)	20.11 (23.74)	4.87 (5.12)	4.76 (4.24)	0.83 (0.96)
0.005	0.25	21.13 (19.93)	19.87 (23.43)	4.96 (5.11)	4.74 (4.14)	0.10 (0.11)
0.0005	0.25	20.64 (19.69)	18.73 (22.14)	4.65 (5.03)	4.65 (4.30)	0.01 (0.01)
0.05	0.5	11.54 (11.53)	11.00 (14.90)	2.45 (2.68)	2.45 (1.93)	0.44 (0.58)
0.005	0.5	11.39 (10.78)	10.65 (13.22)	2.42 (2.50)	2.42 (1.84)	0.05 (0.06)
0.0005	0.5	11.48 (11.28)	11.60 (15.69)	2.49 (2.60)	2.40 (1.85)	0.01 (0.01)
0.05	0.75	7.67 (7.37)	8.15 (11.75)	1.82 (1.92)	1.61 (1.03)	0.32 (0.45)
0.005	0.75	8.05 (7.62)	8.62 (12.58)	1.83 (1.90)	1.70 (1.10)	0.04 (0.06)
0.0005	0.75	7.44 (7.41)	7.08 (10.21)	1.64 (1.70)	1.61 (0.99)	0.00 (0.01)
0.05	0.95	6.25 (5.60)	7.07 (10.29)	1.45 (1.47)	1.31 (0.66)	0.28 (0.39)
0.005	0.95	6.15 (5.92)	6.84 (10.31)	1.44 (1.46)	1.30 (0.65)	0.03 (0.05)
0.0005	0.95	6.01 (5.76)	6.39 (9.69)	1.44 (1.51)	1.31 (0.68)	0.00 (0.00)

Sampled data for three different transmission assumptions and five levels of HHC for one colonised *nurse* starting in a random room in the hospital ward, a hand hygiene efficacy (*γ*) of 0.83. *P*= probability of transmission, *λ*= HHC level, *RO*1= amount of time spent colonised, *RO*2= number of contact moments, *RO*3= number of HCWs or patients made contact with, *RO*4= number of transitions between hospital ward rooms, *RO*5= expected number of HMO transmissions

**Table 7:** Simulated HMO transmissions potential of a colonised nurse in a hospital ward under various assumed transmission rates and hand hygiene compliance levels (between 6 pm and 6 am or on weekends)

Transmission probability	HHC	RO1: Minutes spent colonised (SD)	RO2: Contacts (SD)	RO3: People contacted (SD)	RO4: Room transitions (SD)	RO5: Expected infections (SD)
0.05	0.05	114.30 (89.05)	104.29 (90.80)	16.39 (12.52)	23.14 (21.74)	3.88 (3.26)
0.005	0.05	115.67 (90.79)	104.93 (93.49)	16.44 (12.95)	23.85 (22.56)	0.51 (0.45)
0.0005	0.05	110.68 (87.39)	98.95 (88.37)	15.73 (12.42)	23.11 (22.72)	0.05 (0.04)
0.05	0.25	26.29 (27.74)	27.08 (34.68)	4.00 (4.18)	4.56 (3.96)	0.98 (1.21)
0.005	0.25	28.14 (31.78)	28.35 (37.61)	4.30 (4.36)	4.87 (4.27)	0.13 (0.18)
0.0005	0.25	26.66 (27.13)	26.71 (32.87)	4.13 (4.21)	4.71 (4.25)	0.01 (0.02)
0.05	0.5	13.69 (14.25)	14.73 (22.87)	2.24 (2.32)	2.41 (1.83)	0.53 (0.72)
0.005	0.5	13.58 (14.10)	14.87 (23.00)	2.18 (2.34)	2.42 (1.83)	0.07 (0.11)
0.0005	0.5	14.47 (15.32)	16.10 (23.31)	2.42 (2.56)	2.61 (2.12)	0.01 (0.01)
0.05	0.75	9.66 (9.23)	11.81 (18.08)	1.68 (1.74)	1.67 (1.06)	0.42 (0.59)
0.005	0.75	9.78 (9.31)	11.76 (18.39)	1.61 (1.63)	1.58 (0.94)	0.05 (0.09)
0.0005	0.75	9.55 (9.24)	12.56 (21.62)	1.64 (1.67)	1.58 (0.93)	0.01 (0.01)
0.05	0.95	8.27 (7.90)	9.91 (16.48)	1.39 (1.44)	1.29 (0.63)	0.35 (0.51)
0.005	0.95	8.56 (7.99)	9.94 (16.90)	1.32 (1.38)	1.29 (0.65)	0.05 (0.08)
0.0005	0.95	8.68 (8.42)	10.29 (18.04)	1.32 (1.42)	1.31 (0.69)	0.01 (0.01)

Sampled data for three different transmission assumptions and five levels of HHC for one colonised *nurse* starting in a random room in the hospital ward, a hand hygiene efficacy (*γ*) of 0.83. *P*= probability of transmission, *λ*= HHC level, *RO*1= amount of time spent colonised, *RO*2= number of contact moments, *RO*3= number of HCWs or patients made contact with, *RO*4= number of transitions between hospital ward rooms, *RO*5= expected number of HMO transmissions

**Fig. 4:**
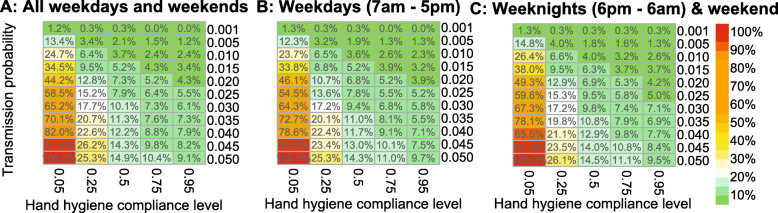
The expected number of transmissions expressed as a percentage of the worst-case scenario. For **a**, **b** and **c**: the highest number of expected transmissions (worst-case scenario) occur for the scenario where the transmission probability is 0.05 and hand hygiene compliance is 0.05. The expected number of transmissions is expressed as a percentage of the worst-case scenario

RO5 is expressed as a percentage RO5 worst-case scenario (*P* = 0.05; *λ* = 0.05) RO5 for subset 1 (*RO*5 = 3.16) and 2 (*RO*5 = 3.88) and the combination of the two (*RO*5 = 3.36) in Fig. 4. This pivoted view of RO5 shows similar changes in the expected numbers of transmissions for both subsets even though the nominal values of RO5 are different. A likely explanation is that during weekdays (Fig. 4:b) increasing hand hygiene from 0.05 to 0.75 has a similar effect as decreasing the transmission probability by a factor 10 (0.05 vs 0.005).

## Discussion

This study identified nurses as a potential super-spreader healthcare worker (HCW) occupation group in a healthcare setting. Nurses have a disproportionately high potential to transmit hand-transmittable harmful microorganisms (HMO) to other HCWs or patients as compared to the other HCW occupation groups. The expected number of transmissions caused by a colonised nurse increases exponentially as the level of hand hygiene compliance (HHC) deteriorates or the transmission probability increases. These results are due to the spatiotemporal behaviour and social mixing patterns of HCWs.

Five risk outcomes were defined to quantify the spatiotemporal effects of varying levels of HHC on the transmission and spread of HMO. These were: 1) the time that a colonised super-spreader is expected to be colonised; 2) the number of contact moments with other HCWs or patients; 3) the number of HCWs or patients encountered; 4) the number of ward rooms frequented while colonised and 5) the expected number of HCWs or patients a super-spreader will transfer microbes to before performing proper hand hygiene. The risk outcomes were quantified for various levels of hand hygiene compliance and probabilities of transmission. The expected change in the number of transmissions for different levels of HHC may encourage approval for healthcare interventions such as increased education and awareness about HHC and strategic accessibility to alcohol dispensers in healthcare settings.

The simulation results are based upon empirical social mixing patterns of HCWs and highlight one colonised nurse’s impact as the super-spreader. These results are applicable when an HMO is transmittable by hand and can be eliminated by hand hygiene using an alcoholic rub. Depending upon the HMO, the probability of transmission may differ, resulting in a change in the expected number of transmissions for various levels of hand hygiene compliance. Such simulations can be used in educational materials to emphasize personal control and responsibility to perform HHC. Normal HHC levels of 50% may deteriorate to 25% during busy periods in a healthcare setting because of reduced healthcare worker capacity or time pressure. The simulation results allow for “what if?” questions to be answered under different assumed levels of HHC and transmission probabilities in terms of the five risk outcomes. HCWs are then able to simulate the impact of the initial assumptions on the expected number of transmissions caused by a super-spreader based on empirical spatiotemporal behaviour and social mixing patterns of HCWs in a real healthcare setting.

The results are consistent with other work done on super-spreaders in healthcare facilities [22]. Our contribution is that we quantified the potential consequences of the spatiotemporal behaviour of HCWs for varying levels of hand hygiene compliance and different transmission probabilities. The simulation results showed that, for the same transmission rates and HHC levels, the number of transmissions is higher during weeknights and weekends. An explanation is that HCWs spent more time with fewer HCWs or patients during weeknights and weekends but had more contact moments for every minute spent colonised. An increase in the time that a super-spreader navigates through the hospital ward results in an increase in the number of encountered HCWs or patients, allowing for more opportunity to transmit the HMO. HHC may vary over time because of varying ward occupancy levels or different days of the week: the simulation results show that for an HMO with a transmission rate of 0.05 and with the average level of HHC of 50% during the week and 25% over the weekend, that the expected number of HCWs and patients to whom a colonised nurse transfers microbes will almost double. Simulation scenarios were identified with equal risk outcomes for different initial conditions. They illustrate that infection prevention and control interventions can use combinations of strategies and bundles of interventions to fight the transmission and spread of HMO to achieve the same results.

The expected number of HCWs or patients to whom a super-spreader will transfer microbes before performing proper hand hygiene (risk outcome 5) is controlled by managing spatiotemporal behaviour (risk outcomes 1–4) and the level of HHC. A possible intervention based upon these results is to limit the number of room transitions, contact and contact duration during periods of low expected levels of HHC. If, for example, on busy Friday evenings the levels of HHC change, a possible preventative intervention might be to optimise the number of HCWs, as well as their routes and logistics according to an algorithm based upon sampled spatiotemporal movement data. Such an algorithm should then specifically be designed to minimise the potential transmission and spread of harmful microorganisms. Risk outcomes can thus be monitored over time, for instance, allowing one to determine the seasonality of trends or the effects of spatiotemporal interventions or policy changes. The five risk outcomes may then be addressed as spatiotemporal safety behaviours in hospital wards and in the formulation of healthcare policy to minimise the transmission of hand-transmittable HMOs.

This study contributes to infection prevention and control by highlighting five risk outcomes essential to describing the possible spread of an HMO on an individual temporal level and the spatial level in a healthcare setting. These insights apply to hand-transmittable HMOs and can be used to develop better informed preventative strategies, for heterogeneous hand hygiene education, feedback, work-place reminders and other interventions.

### Limitations and future work

Our sample is taken over 7 days, giving a unique sample with good coverage for a single week. Differences may exist, however, with other weeks throughout the year and even between years. The data used in this study may further be biased towards HCWs who were diligent in wearing the RFID badges. The data were carefully checked for any inconsistencies; some loss in data quality caused by incorrect room classification because of overlapping RFID reader areas could still be present in the data. This study’s hospital ward is similar to hospital wards found in most healthcare facilities in most aspects. Our results are based upon the sampled RFID tracking data for one specific ward. It is a future challenge to generalise these results to other wards in other hospitals.

The spatial resolution of our data is the room level and an assumption was made regarding the proximity and interaction between people, thus adding uncertainty in the simulation results. The transmission probability may be different during the day than during the evening shifts due to the difference of care provided during different times of the day. Future opportunities include collecting data of a higher spatial resolution that will allow us to identify the proximity between people, within room locations in the hospital and interaction with objects like hand hygiene dispensers and mobile (diagnostic) equipment like computers on wheels. An increase in spatial resolution will enable a more accurate event classification and result in more accurate simulation results. For instance, interaction with a hand hygiene dispenser does not require any assumption about the level of HHC, but only on its efficacy. Interaction of an HCW with objects and equipment inside different architectural designs and room layouts allows one to refine the transmission models, thus improving the transmission scenarios. The spatiotemporal risk outcomes defined should be further investigated to identify the relationship between them. For example, how the expected number of infections change should if the average contact duration contact decreases. These relationships can be used to determine how the risk outcomes should be addressed to reduce the hand transmission of HMOs efficiently.

## Conclusion

This study defines five risk outcomes in terms of the number of contact moments, the duration of contacts and the number of ward rooms frequented while colonised and uses them to quantify the transmission and spread of harmful microorganisms. It shows that nurses are potential super-spreaders of harmful microorganisms due to their spatiotemporal movement and social mixing patterns in a healthcare setting. The expected number of healthcare workers and patients, to whom a super-spreader transfers microbes, increases exponentially as the level of hand hygiene compliance deteriorates. The performed simulations increase our insight into the consequences of varying levels of adherence to spatiotemporally specific healthcare policies such as hand hygiene compliance. The simulations further show that a change in spatiotemporal movement and social mixing patterns of healthcare workers will affect the expected number of transmissions in a closed healthcare setting. The risk outcomes may be further addressed in terms of spatiotemporal safety behaviour in healthcare settings to reduce the spread of HMO. The adherence level is to be further investigated to improve the information to policymakers and further educate healthcare workers about the risks of their spatiotemporal behaviour.

## Supplementary Information

**Additional file 1.** Simulation algorithm.

## Data Availability

The data that support the findings of this study are available from UMCG but restrictions apply to the availability of these data, which were used under license for the current study, and so are not publicly available. Data are however available from the authors upon reasonable request and with permission of UMCG.

## Abbreviations

HCW: Healthcare Worker
HHC: Hand Hygiene Compliance
HMO: Harmful Microorganisms
MRSA: Methicillin-Resistant *Staphylococcus aureus*
RFID: Radio Frequency IDentification
SD: Standard Deviation
SSE: Super-Spreading Events
UMCG: University Medical Center Groningen
WC: Water Closet
