# Supplementary material for: A spatiotemporal simulation study on the transmission of harmful microorganisms through connected healthcare workers in a hospital ward setting
Source: BMC Infect Dis. 2021 Mar 12;21:260. doi: 10.1186/s12879-021-05954-7 (PMC7953685; doi:10.1186/s12879-021-05954-7)
Supplement: Supplementary file 1 — Additional file 1. Simulation algorithm. [file 12879_2021_5954_MOESM1_ESM.docx]

# Additional file 1:

For objective 1, i.e. the identification of a potential super-spreading HCW occupation group ($G$) we performed the following steps:

Step 1.1: Obtain the total time spent in minutes ($\psi)$ and the number of contacts ($M$) between all groups of HCW occupations or patients.

Step 1.2: Rank order HCW occupation groups by $\psi$ and $M$.

Step 1.3: Identify the HCW occupation group with the highest $\psi$ and $M$ and in this sense differs the most from the other HCW occupation group as $G$.

The simulation procedure is performed as follows:

Objective 2

2.1 Obtain sample statistics

Step 2.1.1: Use the sampled data generated by the sensors carried by $G$ to construct a 1-step transition probability matrix ($\boldsymbol{P}$) for the transitions between rooms $R = \{R_{1},R_{2},\ldots,R_{n}\}$.

Step 2.1.2: Obtain the average and standard deviation of the number of HCWs or patients co-occurring with $g$ ∈ $G$ in each room $R_{i}$ as $\omega_{R_{i}}$.

Step 2.1.3: Obtain the average and standard deviation of the number of minutes spent by $G$ in each room as $\psi_{R_{i}}$.

2.2 Simulation

Step 2.2.1 (Workflow A): Select at random a room $R_{i}$ for $i=1,\ldots,n$ as the initial room where $g$ will start the simulation.

Step 2.2.2: Select five equally probable values from a random univariate distribution between 0 and 1 with assigned variables names $u_{1},\ldots,u_{5}$.

Step 2.2.3:

1. (Workflow B): Determine if $g$ successfully performed hand hygiene after entering the room:

$PH=P\left( Successful handhygiene \right)=\lambda\times\gamma$, where we make the assumption that $H\sim N\left( \mu,\sigma\right)$ where $\mu$ and $\sigma$ are as in Table 2 and $\gamma$ is sampled from $H$.Sample a random number $\gamma$ from $H$ until $u_{1}\leq(\gamma*\lambda)$ when the simulation has converged as hand hygiene was performed successfully and the HCW is no longer contagious. Record the number of transitions as $T$.

1. (Workflow C): Simulate the number of HCWs or patients co-occurring in room $R_{i}$:

We assume $\omega_{R_{i}}\sim N\left( \nu,\varphi^{2} \right)$ or $\omega_{R_{i}}\sim Poisson\left( \nu\right)$, where $\nu$ and $\varphi^{2}$ are estimated using the average and variance of $\omega_{R_{i}}$ from the sampled data, respectively. Select a random number from $\omega_{R_{i}}$, using the value $u_{2}$, and round up the results to the nearest integer.

1. (Workflow C): Simulate the number of minutes spent in room $R_{i}$:

We assume that $\psi_{R_{i}}\sim Exp\left( \frac{1}{\eta} \right)$, where η is the sample average of $\psi_{R_{i}},$ estimated from the sampled data. Select a random number from $\psi_{R_{i}}$, using the value $u_{3}$, and round up the results to the nearest integer. Let $m$ be the number of contact moments of $30$ s i.e. $m=\psi_{R_{i}}\times2$.

1. (Workflow D): Simulate the number of $\omega_{R_{i}}$ colonised by $g$ with the HMO:

We assumed each person co-occurring in a room has an independent binomial distribution given by $I\sim Bin(m,P)$ to get colonised by $g$ over $m$ contact moments with and transmission probability $P$. We assume that the successful transmission during the $m$ contact moments for each of the $\omega_{R_{i}}$ co-occurring HCWs or patients is independent, which is why the expected number of HCWs or colonised during a the co-occurrence in room $R_{i}$ ($IN$) can be estimated as $E\left[ IN \right]=\sum_{j=1}^{\omega_{R_{i}}} \left\{ 1-P(I=0) \right\}=\omega_{R_{i}}\times\left( 1-{(1-P)}^{m} \right)$.

1. (Workflow A): Determine the next room after the following transition:

Use $u_{5}$ as input for the inverse cumulative probability distribution of the transition probability matrix $\boldsymbol{P}$ found by taking the cumulative sum on the row of $\boldsymbol{P}$ corresponding to current room $R_{i}$.

1. Three cumulative measures during simulation are collected while $g$ is colonised: the time spent in minutes ($\psi_{c}$), $\omega_{R_{i}}$ co-occurring with $g$ ($\omega_{c}$) and the expected number of HCWs or patients colonised by $g$ (${IN}_{c}$).
2. Repeat Step 2.2.3
